# The Protective Effect of Total Flavones from* Rhododendron simsii* Planch. on Myocardial Ischemia/Reperfusion Injury and Its Underlying Mechanism

**DOI:** 10.1155/2018/6139372

**Published:** 2018-01-09

**Authors:** Sheng-Yong Luo, Qing-Hua Xu, Gong Peng, Zhi-Wu Chen

**Affiliations:** ^1^Department of Pharmacology, Anhui Medical University, Hefei, Anhui 230032, China; ^2^Anhui Academy of Medical Sciences, Hefei, Anhui 230061, China; ^3^Department of Pharmacy, The First Affiliated Hospital of Anhui Medical University, Hefei, Anhui 230032, China

## Abstract

**Objectives:**

Total flavones from* Rhododendron simsii* Planch. (TFR) are the effective part extracted from the flowers of* Rhododendron simsii* Planch. and have obvious protective effects against cerebral ischemic or myocardial injuries in rabbits and rats. However, their mechanism of cardioprotection is still unrevealed. Therefore, the present study was designed to investigate the effect of TFR on myocardial I/R injury and the underlying mechanism.

**Methods:**

TFR groups were treated by gavage once a day for 3 days at a dose of 20, 40, and 80 mg/kg, respectively, and then the model of myocardial I/R injury was established. Myocardial infarction, ST-segment elevation, and the expression of UTR, ROCK1, ROCK2, and p-MLC protein in rat myocardium were determined at 90 min after reperfusion. UTR siRNA* in vivo *transfection and competition binding assay method were used to study the relationship between the protective effect of TFR and UTR.

**Results:**

The expression of UTR protein markedly decreased in myocardium of UTR siRNA transfection group rats. TFR could significantly reduce the infarct size and inhibit the increase of RhoA activity and ROCK1, ROCK2, and p-MLC protein expressions both in WT and UTR knockdown rats. The reducing rate of TFR in myocardial infarction area, RhoA activity, and ROCK1, ROCK2, and p-MLC protein expressions in UTR knockdown rats decreased markedly compared with that in WT rats. In addition, TFR had no obvious effect on the increase of ΣST in UTR knockdown rats in comparison with that in model group. In particular, TFR could significantly inhibit the combination of [^125^I]-hu-II and UTR, and IC_50_ was 0.854 mg/l.

**Conclusions:**

The results indicate that the protective effect of TFR on I/R injury may be correlated with its blocking UTR and the subsequent inhibition of RhoA/ROCK signaling pathway.

## 1. Introduction

Urotensin-II (U-II) was initially isolated from the goby urophysis in the caudal portion of the teleost fish. Subsequent studies have shown that homologs of U-II were present in all mammals acting as a vasoactive peptide [1]. Both U-II and its receptor (UTR), an orphan G protein-coupled receptor 14, are widely expressed throughout the body, particularly in the cardiovascular system [2]. Large amount of data indicated that the level of plasma human U-II and tissue expression levels of human U-II and UTR were upregulated in various cardiovascular diseases such as ischemia [3], heart failure [4], atherosclerosis [5], hypertension [6, 7], chronic hypoxia [8], and myocardial infarction [9]. Furthermore, the serum human U-II concentration was positively correlated with the degree of myocardium injury in a rat myocardial infarction model [10]. It was also found that RhoA/Rho-kinase (Rho-associated coiled-coil-containing protein kinase, ROCK) signaling pathway played an important role in a variety of cardiovascular diseases [11]. Blocking this pathway could inhibit the vascular tone and protect myocardial I/R injury [12, 13]. Moreover, some studies have proven the involvement of RhoA/ROCK pathway in the human U-II-induced physiological and pathological processes, such as heart contraction, arterial smooth muscle cell proliferation, and cardiac diastolic dysfunction [14]. Our previous study has shown that myocardial I/R injury may upregulate the UTR expression in myocardium of rats and UTR antagonist (SB-710411) could significantly reduce the cardiac I/R injury via the inhibition of RhoA/ROCK signaling pathway [15], which points to the fact that RhoA/ROCK pathway is also engaged in the human U-II-induced myocardial I/R injury. Therefore, the hU-II/UT receptor system might be a promising pharmacological target in the treatment of ischemic heart disease.

Flavonoids, which are effective ingredients in many Chinese herbs and are commonly found in natural plants, exert pharmacological functions in various biological activities, including vasorelaxing and anti-inflammatory effect as well as protective effect against myocardial I/R injury [16].* Rhododendron simsii* Planch. is a traditional Chinese medicine that has been used in treatment of bronchitis in China for thousands of years. Total flavones from* Rhododendron simsii* Planch. (TFR) are the effective part extracted from the flowers of* Rhododendron simsii* Planch. consisting of hyperin, quercetin, matteucinol, and rutin [17, 18]. Our previous studies have shown that TFR has obvious protective effects against cerebral ischemic or myocardial injuries in rabbits and rats [19, 20]. However, its mechanism of cardioprotection is still unrevealed. Thus, the present study was designed to investigate the effect of TFR on myocardial I/R injury and the underlying mechanism. Specifically, the main focus is on the relationship between TFR and UTR/RhoA/ROCK pathway.

## 2. Materials and Methods

### 2.1. Sample Preparation

*Rhododendron simsii* Planch. was dried and then milled. The total flavonoids were extracted according to a previously reported method [21]. In brief, air-dried aerial parts were inserted into a Soxhlet apparatus.* Rhododendron simsii* Planch. was exhaustively extracted sequentially with 250 mL petroleum ether, dichloromethane, acetonitrile, ethyl acetate, methanol, n-butanol, and H_2_O. The organic extracts were dried over anhydrous magnesium sulfate, and the solvent was removed in vacuo (40°C). The water extract was concentrated in vacuo (40°C) and lyophilized. The total flavonoid extracts were dissolved in MeOH prior to analysis and the content was determined by UV-spectrophotometry.

### 2.2. Reagents

SB-710411 underwent custom synthesis by GL Biochem Ltd. (Shanghai, China). G-LISA RhoA Activation Assay Biochemistry Kit^TM^ was purchased from Cytoskeleton (Denver, USA). UTR antibody (1 : 200) was obtained from WuXi AppTec (San Diego, USA); mouse monoclonal antibodies against ROCK1 and ROCK2 (1 : 1000) were bought from Nanjing Enogene Biological Co. (Nanjing, China). Mouse monoclonal antibodies against myosin light chain (MLC) and phosphorylated MLC (p-MLC) were derived from Cell Signaling Technology Inc. (Beverly, MA, USA). 2,3,5-Triphenyltetrazolium chloride (TTC) and Evans Blue were acquired from Sigma-Aldrich (St. Louis, MO, USA). Rat UTR siRNA (sense, 5′-UGGCCUCCAUGUACGUCUATT-3′; antisense, 5′-UAGACGUACAUGGAGGCCATT-3′) and negative control siRNA (sense, 5′-UUCUCCGAACGUGUCACGUTT-3′; antisense, 5′-ACGUGACACGUUCGGAGAATT-3′) were supplied by Shanghai GenePharma Co., Ltd. (Shanghai, China). Entranster™-*in vivo* RNA transfection reagent was gained from Engreen Biosystem Co., Ltd. (Beijing, China). Na^125^I was purchased from PerkinElmer Co., Ltd. (Shanghai, China).

### 2.3. Animals

Male Sprague-Dawley rats weighing 250–300 g were purchased from the Anhui Medical University Animal Center. All rats were housed in a room temperature of 22 ± 4°C and kept on a 12 hr light/dark cycle with free access to food and water. Animal care and experimental protocols were approved by the committee on the Ethics of Animal Experiments of Anhui Medical University, in accordance with the Guide for the Care and Use of Laboratory Animals of the National Institutes of Health (NIH Publication, 8th edition, 2011).

### 2.4. UTR siRNA In Vivo Transfection

To knockdown the UTR expression in rat heart, UTR siRNA* in vivo* transfection was performed by injecting a predesigned siRNA specifically targeting UTR (5′-UGGCCUCCAUGUACGUCUATT-3′; 5′-UAGACGUACAUGGAGGCCATT-3′). A scrambled siRNA was used as a negative control (5′-UUCUCCGAACGUGUCACGUTT-3′; 5′-ACGUGACACGUUCGGAGAATT-3′). The UTR siRNA or scrambled siRNA (50 ug) was diluted in 25 uL of Entranster-*in vivo* reagent and 10% glucose mixture according to the manufacturer's instructions. Each rat was intravenously injected with the siRNA mix (the final dose was 250 ug/ml) in a volume of 200 uL. After 72 h of UTR siRNA* in vivo* transfection, successful knockdown of UTR in rat heart was assessed by Western blot [22].

### 2.5. Experimental Protocol

Sprague-Dawley rats (WT and UTR knockdown rats) were randomly divided into the following 7 groups: sham, model, nifedipine 5.4 mg/kg, SB-710411 2.0 *μ*g/kg, and TFR 20, 40, and 80 mg/kg groups. The rats in nifedipine and SB-710411 groups were intravenously injected (i.v.) daily for 3 days at a dose of 5.4 mg/kg and 2.0 *μ*g/kg, respectively, and TFR groups were treated by gavage once a day for 3 days at a dose of 20, 40, and 80 mg/kg, respectively.

### 2.6. Surgical Procedures

The surgical protocol was carried out in accordance with the previous method with slight modifications [23]. In brief, Sprague-Dawley rats were anaesthetized with pentobarbital (40 mg/kg) by intraperitoneal injection. The fourth and fifth ribs on the left side of the chest were cut to perform the thoracotomy and the pericardium was incised. The heart was gently exteriorized and a 5/0 silk suture was placed around the left anterior descending (LAD) coronary artery, which was 1-2 mm under the left auricle. The suture then was ligated and the ends of this ligature were passed through a small plastic tube to form a snare. After 30 min of ischemia, the snare was removed gently, allowing reperfusion for 90 min. Ischemia was confirmed by ST-segment elevation on the electrocardiogram and visible color changes in the ischemic myocardial region. Rats in the sham group underwent the same surgical procedures with the exception of LAD ligation. After the I/R protocol, the animals were sacrificed with an overdose of 10% chloral hydrate (500 mg/kg,* i.v*.). The hearts were harvested for the evaluation of infarct size and some related signal proteins expression.

### 2.7. Measurement of Infarct Size

At the end of 90 min reperfusion, the LAD of rats was reoccluded (except the sham group) and 0.5% Evans Blue was injected into the aortic cannula. The hearts were then harvested and were frozen at −20°C in a freezer. After removing the right ventricle, the left ventricles were cut into five 2 mm transverse slices from the apex to the base and incubated in 1% TTC in phosphate buffer at 37°C for 20 min. The nonischemic area was stained blue, the ischemic area was stained light red, and the infracted area was not stained and presented as pale [24]. Slices were photographed subsequently and measured to delineate the area of infarct size (IS, TTC-negative) and area at risk (AAR, TTC stained). Myocardial infarct size was calculated as the ratio of IS/AAR by computerized planimetry using ImageJ version 1.6 (National Institutes of Health, Bethesda, MD, USA) [15, 25].

### 2.8. Western Blotting

The total proteins in the left ventricular tissues of rat hearts were extracted according to the method described previously with modifications [15, 26]. Briefly, the tissue was homogenized and lysed with RIPA lysis buffer (1 mM sodium orthovanadate, 50 mM Tris HCl, 1% Triton X-100, 150 mM NaCl, 1 mM glycerophosphate, 1 mM DTT, and protease inhibitor) on ice for 30 min and then centrifuged at 12,000*g* for 10 min at 4°C to separate soluble fractions from insoluble ones. The protein concentration in the supernatants was measured spectrophotometrically at a 562 nm wavelength using bicinchoninic acid (BCA) protein assay kit. The total protein (30 *μ*g) was loaded and separated by 10% SDS-polyacrylamide gel electrophoresis (PAGE) and then transferred to polyvinylidene difluoride (PVDF) membranes. After blocking with buffer containing 5% skimmed milk in TBS-T containing 10 mmol/L Tris-HCl (pH 6.8), 150 mmol/L NaCl, and 0.05% Tween 20, the membranes were incubated overnight at 4°C with the primary antibodies of UTR, ROCK1, ROCK2, or p-MLC/MLC. The membranes were washed three times with TBST buffer for 15 minutes and subsequently incubated in diluted appropriate secondary antibody for 1 h at 37°C. Relative intensity of the band was quantified by densitometry of radioautograph films.

### 2.9. RhoA Activity

Absorbance-based G-LISA RhoA Activation Assay Biochemistry Kit was applied to determine the RhoA activity in rat myocardium [15, 27]. Frozen tissues were homogenized in lysis buffer, and the protein concentration was detected in accordance with the manufacturer's instructions. RhoA activity is determined by measuring absorbance at 490 nm with microplate spectrophotometer after indirect immunodetection.

### 2.10. Competition Binding Assay

The hearts of WT rats were rapidly removed and the tissues from the left ventricle were immediately placed on ice in cold buffer (50 mM Tris-HCl, 1 mM EGTA, and 5 mM MgCl_2_, pH 7.4). The tissues were minced with scissors and homogenized in ice-cold Tris-HCl buffer (pH 7.5 at room temperature). The homogenates were centrifuged at 48000 ×g for 20 min at 4°C. Membrane fractions were resuspended in buffer followed by incubation at 37°C for 10 min to remove endogenous ligands. The homogenates were centrifuged again and resuspended in assay buffer. Competition binding assays were performed using the scintillation proximity assay (SPA) method. Membranes were precoupled to wheatgerm-agglutinin-coated SPA beads. Binding conditions (200 ml final volume) consisted of 10 mg membrane protein, 0.4 mg SPA beads, and 0.3 nM [^125^I]-hU-II in the presence of varying concentrations of TFR (2.43 × 10^−3^–10^3^ mg/ml) in assay buffer (20 mM Tris-HCl, pH 7.4, 5 mM MgCl_2_, and 0.05% BSA). After incubation for 2 hrs at room temperature, binding reactions were stopped by rapid filtration under reduced pressure through GF/C filters [28]. Nonspecific binding was determined with 1 *μ*M unlabeled hU-II. Assay plates were sealed, shaken gently for 1 h at room temperature, centrifuged at 2000 ×g for 10 min, and then counted using *γ*-counter to quantify residual radioactivity. Competition binding curves were plotted using GraphPad Prism 5.0 software.

### 2.11. Statistical Analysis

All numerical data are presented as means ± SD. Paired* t*-tests were used to compare the differences between TFR (or other drugs) and control groups in WT or UTR knockdown rats. The statistically significant differences in the influence degree of TFR (or other drugs) for each detection index compared with those in control group between WT and UTR knockdown rats were assessed by one-way analysis of variance or nonparametric test. *p* < 0.05 was considered statistically significant.

## 3. Results

### 3.1. Expression of UTR Protein in Rat Myocardium after UTR siRNA In Vivo Transfection

**Figure 1:**
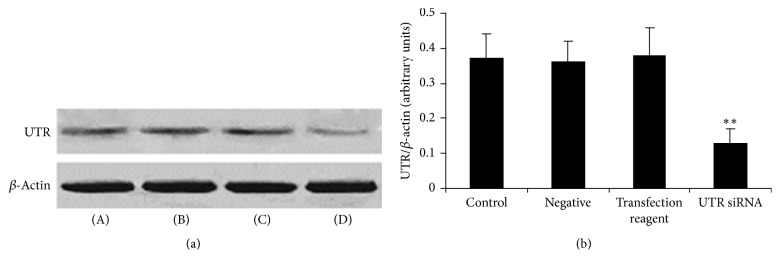
*The expression of UTR protein in rat myocardium after UTR siRNA in vivo transfection*. (a) UTR protein was detected by Western blot. *β*-Actin was used as the loading control. (b) Densitometric quantification of UTR protein expression. (A): control; (B): negative; (C): transfection reagent; and (D): UTR siRNA. Results are expressed as mean ± SD (*n* = 5, each group). ^*∗∗*^*p* < 0.01 versus control group.

As shown in Figure 1, compared with that in control group (WT rats, 0.37 ± 0.07), the expression of UTR protein significantly decreased in rat myocardium of UTR siRNA transfection group (0.13 ± 0.04). However, the negative (scrambled siRNA) and transfection reagent had no obvious effect on the expression of UTR protein in rat myocardium (0.36 ± 0.06 and 0.38 ± 0.08, resp.).

### 3.2. Effect of TFR on I/R Injury-Induced Myocardial Infarction

**Figure 2:**
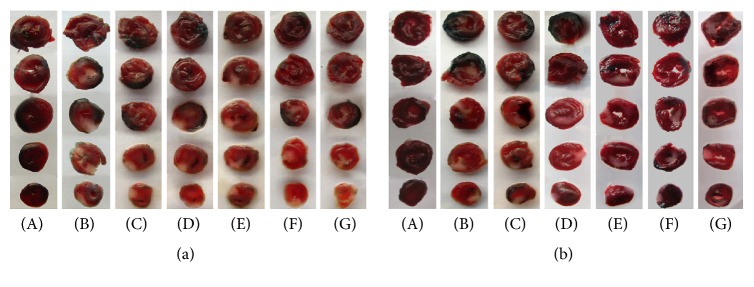
*Evans Blue and 2,3,5-triphenyltetrazolium chloride (TTC) double staining in each group's rats*. Evans Blue and 2,3,5-triphenyltetrazolium chloride (TTC) double staining was used to determine the ischemic size and infarction size. (a) WT rats myocardial slice stained by Evans Blue and TTC. (b) UTR knockdown rats myocardial slice stained by Evans Blue and TTC. The normal area was stained blue, the area at risk (ARR) was stained red, and the area of infract size (IS) was stained pale white. (A): sham; (B): model; (C): 5.4 mg/kg nifedipine; (D): 2.0 *μ*g/kg SB-710411; and (E)~(G): 20, 40, and 80 mg/kg TFR.

**Figure 3:**
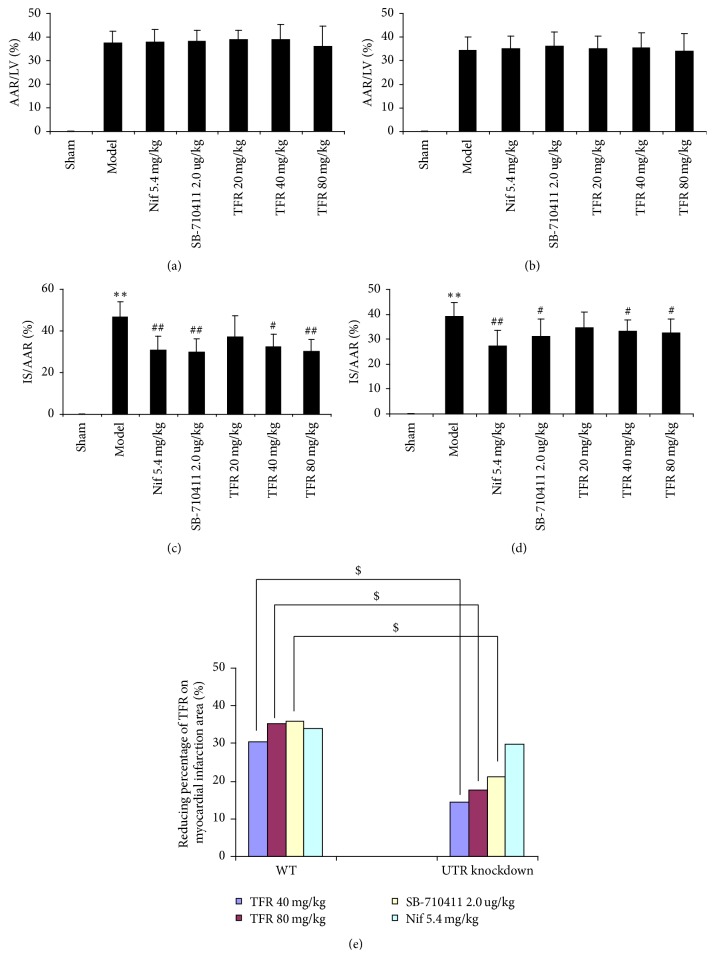
*ARR and IS in each group's rats*. (a) Effect of TFR on the ARR in WT rat. (b) Effect of TFR on the ARR in UTR knockdown rat. ARR was expressed as a percentage of the left ventricular area (AAR/LV). (c) Effect of TFR on the IS in WT rats. (d) Effect of TFR on the IS in UTR knockdown rats. The IS was normalized by expressing it as a percentage of the ARR (IS/AAR). (e) Reducing percentage of TFR on myocardial infarction area. Results are expressed as mean ± SD (*n* = 8, each group). ^*∗∗*^*p* < 0.01 versus the sham group; ^#^*p* < 0.05 and ^##^*p* < 0.01 versus the model group; ^$^*p* < 0.05 versus WT rats.

As shown in Figures 2 and 3, neither area at risk (AAR) nor infarct size (IS) was observed in the sham group in both WT and UTR knockdown rats. I/R injury induced significant myocardial infarct in the model group by measurement of the I/S/ARR ration, and there were obvious differences between WT and UTR knockout rats in terms of infarct area (46.74% ± 7.34% in WT rats; 39.22% ± 5.46% in UTR knockdown rats). The IS/AAR ratio was significantly reduced in the TFR 40 and 80 mg/kg groups (32.37% ± 6.20% and 30.04% ± 5.87% in WT rats; 33.39% ± 4.33% and 32.40% ± 5.78% in UTR knockdown rats) compared with that in the model group (*p* < 0.05; *p* < 0.05 or *p* < 0.01). However, the reducing percentage of TFR (40 and 80 mg/kg) on myocardial infarction area in UTR knockdown rats decreased markedly compared with that in WT rats (*p* < 0.05).

### 3.3. Effect of TFR on ST-Segment Change after Myocardial I/R Injury

**Figure 4:**
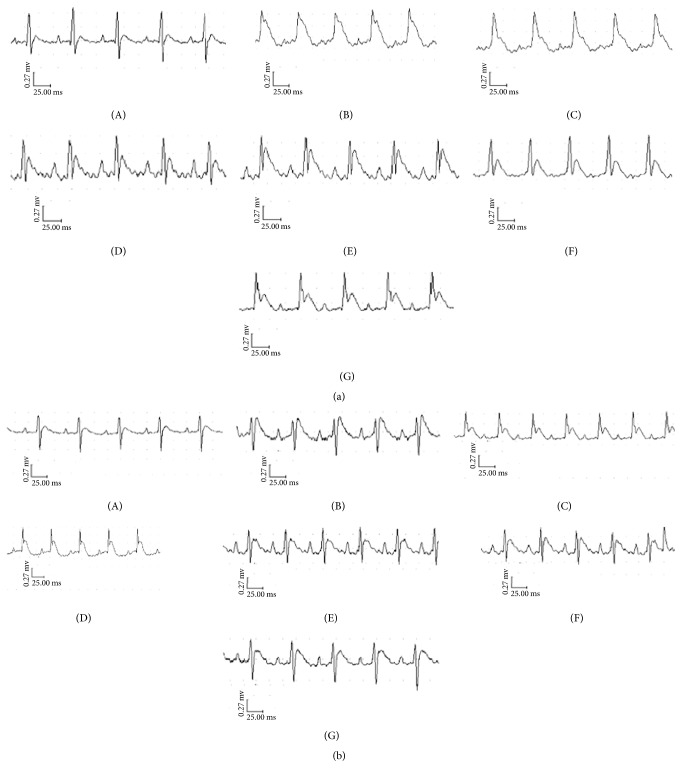
*The traces of the ECG in each group*. Rats were subjected to 30 min of myocardial ischemia followed by 90 min of reperfusion, and electrocardiogram (ECG) was measured. (a) The traces of the ECG in each group of WT rats. (b) The traces of the ECG in each group of UTR knockdown rats. (A): sham; (B): model; (C): 5.4 mg/kg nifedipine; (D): 2.0 *μ*g/kg SB-710411; and (E)~(G): 20, 40, and 80 mg/kg TFR.

**Figure 5:**
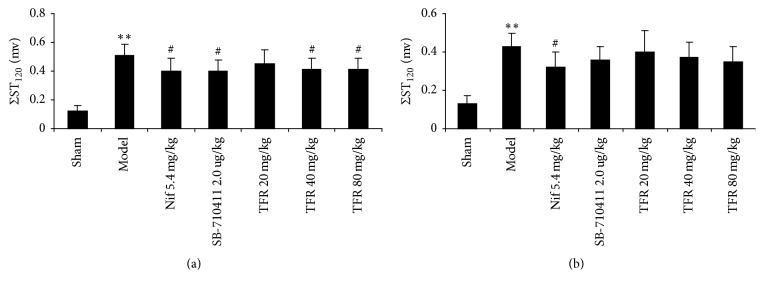
Σ*ST elevation in each group*. (a) Effect of TFR on ΣST elevation in WT rats. (b) Effect of TFR on ΣST elevation in UTR knockdown rats. Results are expressed as mean ± SD (*n* = 8, each group) ^*∗∗*^*p* < 0.01 versus the sham group; ^#^*p* < 0.05 versus the model group.

As shown in Figures 4 and 5, I/R injury caused severe ST-segment (ΣST) elevation in the model group in both WT and UTR knockdown rats. Treatment with 40 and 80 mg TFR could significantly attenuate the increase of ΣST in WT rats (*p* < 0.05 versus the model group). However, TFR had no obvious effect on the increase of ΣST in UTR knockdown rats (*p* > 0.05 versus the model group).

### 3.4. Effect of TFR on RhoA Activity

**Figure 6:**
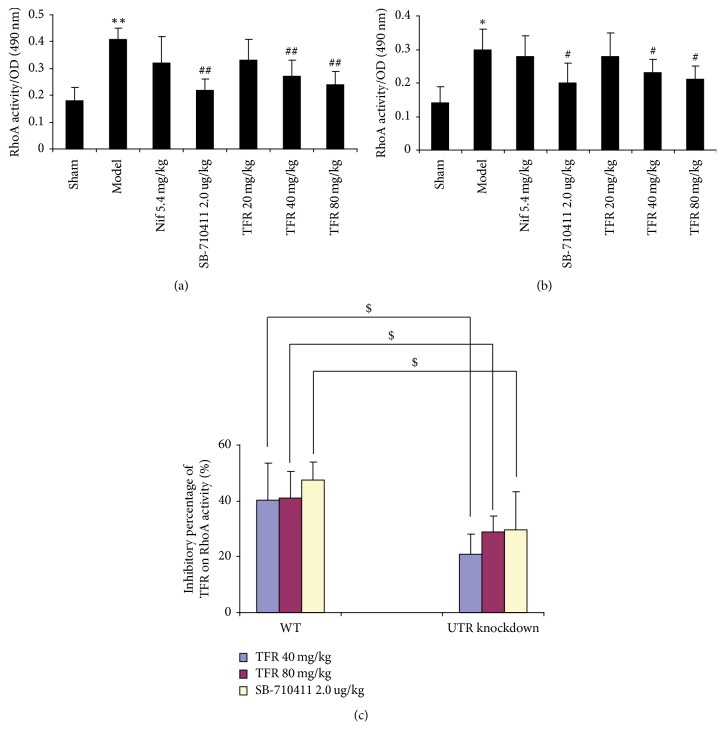
*Effect of TFR on RhoA activity*. The activity of RhoA in myocardium was detected by luminescence-based G-LISA™ assay. (a) The RhoA activity in each group of WT rats. (b) The RhoA activity in each group of UTR knockdown rats. (c) The inhibitory percentage of TFR on RhoA activity. Results are expressed as mean ± SD (*n* = 6, each group). ^*∗∗*^*p* < 0.01 versus the sham group; ^#^*p* < 0.05 and ^##^*p* < 0.01 versus the model group; ^$^*p* < 0.05 versus WT rats.

RhoA activity in the myocardium was detected using G-LISA method. As shown in Figure 6, RhoA activity in the myocardium of the model group (0.41 ± 0.04 in WT rats; 0.30 ± 0.06 in UTR knockdown rats) increased significantly compared with that in the sham group (0.18 ± 0.05 in WT rats; 0.14 ± 0.05 in UTR knockdown rats) in both WT rats and knockdown rats. Treatment with 40 and 80 mg TFR could obviously inhibit the increase of RhoA activity. The activity was reduced to 0.27 ± 0.06 and 0.24 ± 0.06 in WT rats and 0.23 ± 0.04 and 0.21 ± 0.04 in UTR knockdown rats, respectively (*p* < 0.01, in WT rats; *p* < 0.05, in UTR knockdown rats). However, the inhibitory percentage of TFR (40 and 80 mg/kg) on RhoA activity in UTR knockdown rats decreased markedly compared with that in WT rats (*p* < 0.05).

### 3.5. Effect of TFR on Expressions of ROCK1 and ROCK2 Proteins

**Figure 7:**
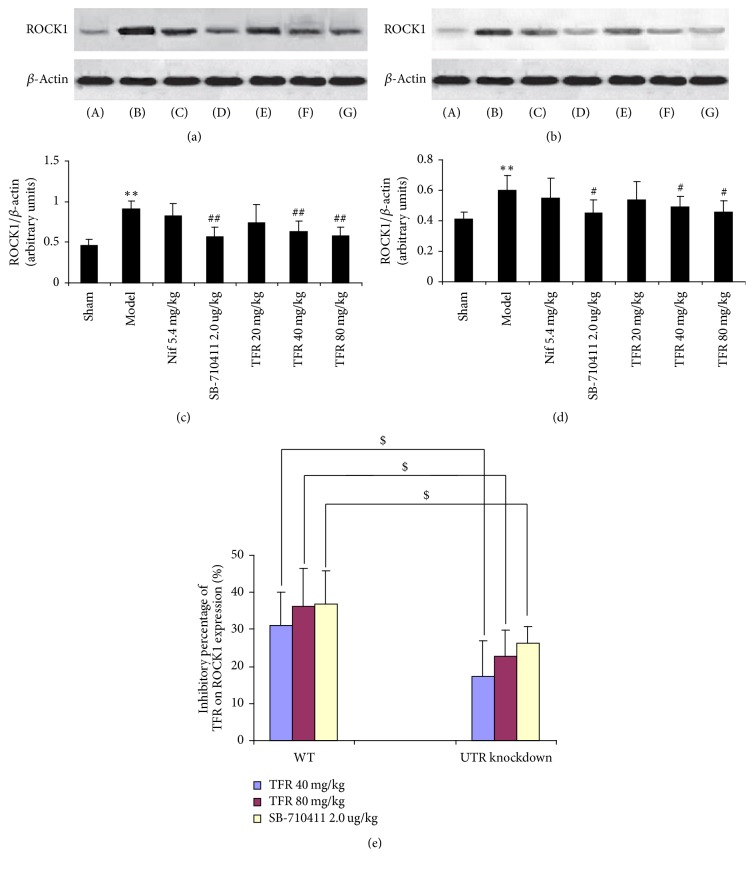
*Effect of TFR on expressions of ROCK1 protein*. (a) ROCK1 protein of WT rats was detected by Western blot method. *β*-Actin was used as the loading control. (b) ROCK1 protein of UTR knockdown rats was detected by Western blot method. *β*-Actin was used as the loading control. (c) Densitometric quantification of ROCK1 protein expression in WT rats. (d) Densitometric quantification of ROCK1 protein expression in UTR knockdown rats. (e) The inhibitory percentage of TFR on ROCK1 protein expression. (A): sham; (B): model; (C): 5.4 mg/kg nifedipine; (D): 2.0 *μ*g/kg SB-710411; and (E)~(G): 20, 40, and 80 mg/kg TFR. Results are expressed as mean ± SD (*n* = 6, each group). ^*∗∗*^*p* < 0.01 versus the sham group; ^#^*p* < 0.05 and ^##^*p* < 0.01 versus the model group; ^$^*p* < 0.05 versus WT rats.

**Figure 8:**
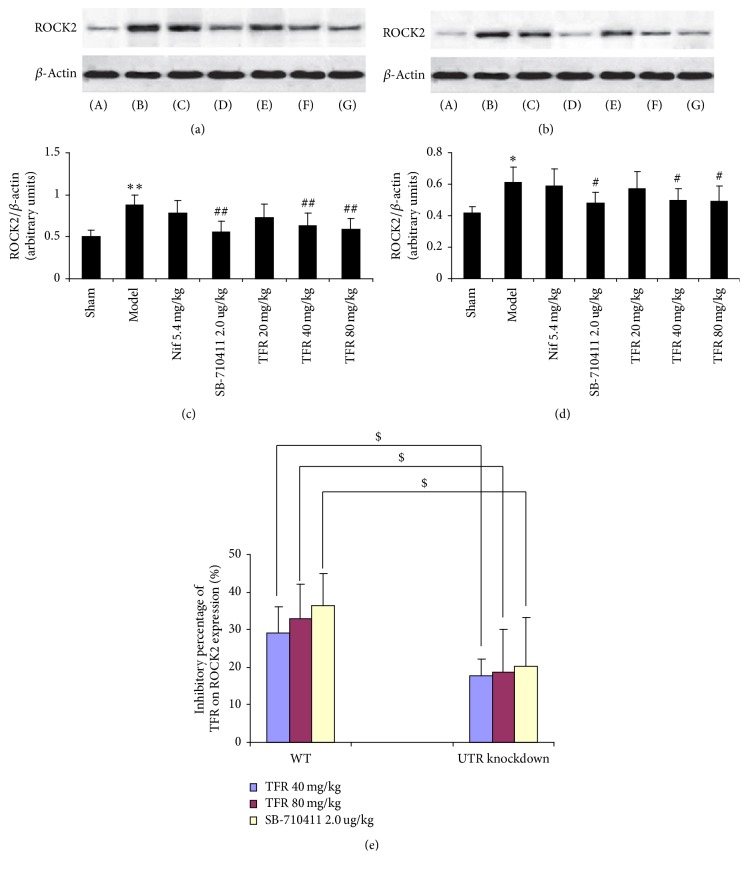
*Effect of TFR on expressions of ROCK2 protein*. (a) ROCK2 protein of WT rats was detected by Western blot method. *β*-Actin was used as the loading control. (b) ROCK2 protein of UTR knockdown rats was detected by Western blot method. *β*-Actin was used as the loading control. (c) Densitometric quantification of ROCK2 protein expression in WT rats. (d) Densitometric quantification of ROCK2 protein expression in UTR knockdown rats. (e) The inhibitory percentage of TFR on ROCK2 protein expression. (A): sham; (B): model; (C): 5.4 mg/kg nifedipine; (D): 2.0 *μ*g/kg SB-710411; and (E)~(G): 20, 40, and 80 mg/kg TFR. Results are expressed as mean ± SD (*n* = 6, each group). ^*∗∗*^*p* < 0.01 versus the sham group; ^#^*p* < 0.05 and ^##^*p* < 0.01 versus the model group; ^$^*p* < 0.05 versus WT rats.

As indicated in Figures 7 and 8, the protein expressions of ROCK1 and ROCK2 in rat myocardium were detected in the sham group (0.46 ± 0.08 and 0.50 ± 0.08 in WT rats; 0.41 ± 0.05 and 0.42 ± 0.04 in UTR knockdown rats). Myocardial I/R injury dramatically induced increase of both ROCK1 and ROCK2 expressions (0.91 ± 0.10 and 0.88 ± 0.12 in WT rats; 0.60 ± 0.10 and 0.61 ± 0.10 in UTR knockdown rats) (*p* < 0.01 and *p* < 0.05). TFR 40 and 80 mg/kg could significantly inhibit the increase of ROCK1 and ROCK2 expressions (ROCK1: 0.63 ± 0.13 and 0.58 ± 0.11 in WT rats and 0.49 ± 0.07 and 0.46 ± 0.07 in UTR knockdown rats; ROCK2: 0.63 ± 0.15 and 0.59 ± 0.13 in WT rats and 0.50 ± 0.07 and 0.49 ± 0.10 in UTR knockdown rats) compared with those in the model group (*p* < 0.01 in WT rats; *p* < 0.05 in UTR knockdown rats). However, the inhibitory percentage of TFR (40 and 80 mg/kg) on ROCK1 and ROCK2 protein expressions in UTR knockdown rats decreased evidently compared with that in WT rats (*p* < 0.05).

### 3.6. Effect of TFR on Expression of p-MLC/MLC Protein

**Figure 9:**
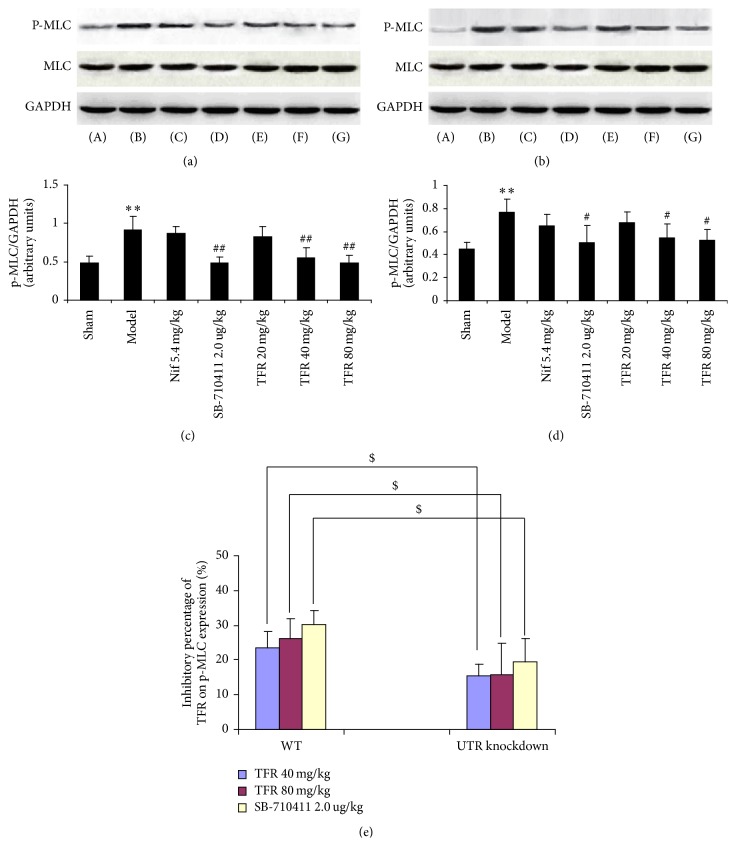
*Effect of TFR on expression of p-MLC/MLC protein*. (a) p-MLC/MLC protein in WT rats was detected by Western blot. *β*-Actin was used as the loading control. (b) p-MLC/MLC protein in UTR knockdown rats was detected by Western blot. *β*-Actin was used as the loading control. (c) Densitometric quantification of p-MLC protein expression in WT rats. (d) Densitometric quantification of p-MLC protein expression in UTR knockdown rats. (e) The inhibitory percentage of TFR on p-MLC protein expression. (A): sham; (B): model; (C): 5.4 mg/kg nifedipine; (D): 2.0 *μ*g/kg SB-710411; and (E)~(G): 20, 40, and 80 mg/kg TFR. Results are expressed as mean ± SD (*n* = 6, each group). ^*∗∗*^*p* < 0.01 versus the sham group; ^#^*p* < 0.05 and ^##^*p* < 0.01 versus the model group; ^$^*p* < 0.05 versus WT rats.

As demonstrated in Figure 9, the protein expression of p-MLC in rat myocardium was detected in the sham group (0.48 ± 0.09 in WT rats; 0.45 ± 0.06 in UTR knockdown rats). Myocardial I/R injury apparently led to increase of p-MLC protein expression (0.91 ± 0.18 in WT rats; 0.77 ± 0.11 in UTR knockdown rats) (*p* < 0.01 and *p* < 0.05). TFR 40 and 80 mg/kg could significantly inhibit the increase of p-MLC protein expression (0.55 ± 0.13 and 0.49 ± 0.10 in WT rats; 0.55 ± 0.12 and 0.53 ± 0.09 in UTR knockdown rats) compared with that in the model group (*p* < 0.01 in WT rats; *p* < 0.05 in UTR knockdown rats). However, the inhibitory percentage of TFR (40 and 80 mg/kg) on p-MLC protein expression in UTR knockdown rats decreased markedly compared with that in WT rats (*p* < 0.05).

### 3.7. Effect of TFR on the Combination of [^125^I]-hu-II and UTR

**Figure 10:**
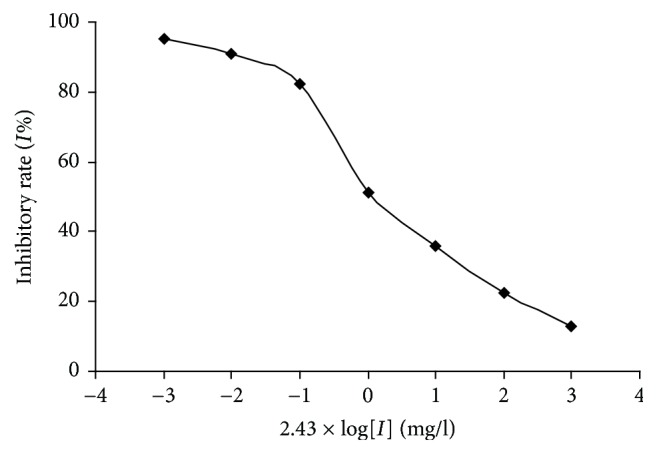
*Effect of TFR on the combination of [* ^125^ *I]-hu-II and UTR*. Competition binding curves were plotted using GraphPad Prism 5.0 software. The semilogarithmic graph of dose response about the inhibiting effect of TFR on the combination of [^125^I]-hu-II and UTR in rat myocardium.

Competition binding assay method was employed to detect the inhibitory effect of TFR on the combination of [^125^I]-hu-II and UTR. As revealed in Table 1 and Figure 10, TFR could significantly block the combination of [^125^I]-hu-II and UTR, and IC_50_ was 0.854 mg/l.

## 4. Discussion 

Urotensin-II and its receptor are highly expressed in the cardiovascular system and play a crucial role in cardiovascular homeostasis [29]. A lot of evidences have shown that hU-II/UTR system is upregulated in pathological conditions such as atherosclerosis, heart failure, hypertension, and ischemic heart disease. Our previous study has also proven that cardiac I/R injury could induce the upregulation of UTR protein expression in rat myocardium, and UTR antagonist (SB-710411) could significantly reduce this injury, which indicates that UTR may have a pathological role in myocardial I/R injury, and the antagonism on UTR may be a novel therapeutic target.

In this study, we have found that TFR has a significant protective effect on the cardiac I/R injury in WT rats, as indicated by attenuation of the increase of ΣST in ECG and reduced infarct size in its cardioprotection role, conforming with the previous report. Furthermore, as revealed by the UTR siRNA* in vivo* transfection and competition binding assay method in the determination of the relationship between protective effect of TFR and UTR, the expression of UTR protein significantly decreased in rat myocardium of UTR siRNA transfection group, and TFR could also significantly reduce the IS/AAR ratio in UTR knockdown rats compared with that in the model group. However, the reducing percentage of TFR on myocardial infarction area in UTR knockdown rats decreased markedly in comparison with that in WT rats. In addition, TFR had no obvious effect on the increase of ΣST in UTR knockdown rats compared with that in model group. Particularly, TFR could significantly inhibit the combination of [^125^I]-hu-II and UTR, and IC_50_ was 0.854 mg/l. The above results suggest that the protective effect of TFR on cardiac I/R injury may be associated with its blocking UTR.

RhoA/ROCK is an important intracellular signaling transduction pathway associated with various pathological conditions [30, 31]. RhoA, one of small guanosine-5′-triphosphate-binding proteins, exhibits GDP/GTP-binding activity and GTPase activity, cycling between a GDP-bound inactive (GDP-Rho) state and a GTP-bound active state (GTP-Rho). ROCK is one of the best characterized effectors of small GTPase RhoA, which belongs to the AGC (protein kinases A, G, and C) family of serine/threonine protein kinases. As a major downstream effector of RhoA, ROCK promotes actin-myosin-mediated contractile force generation by phosphorylating a variety of downstream target proteins and thus plays an important role in many cardiovascular pathogeneses, such as arterial hypertension, atherosclerosis, heart attack, vascular remodeling, and myocardial hypertrophy [32, 33]. ROCK has two isoforms, ROCK1 and ROCK2, which account for 65% overall identity and 92% identity in the kinase domain, respectively. It has been suggested that the phosphorylation of myosin light chain (MLC), one major downstream substrate of ROCK, is involved in the regulation of cardiac function [34]. Overexpression of both ROCK1 and ROCK2 may increase MLC phosphorylation and then promote the actin filament cross-linking activity of myosin II, resulting in muscle contraction, which in turn increases cardiovascular load. Inhibition of ROCK can reduce blood pressure and boost heart function through decreasing the MLC phosphorylation [35].

It has been confirmed that both ROCK1 and ROCK2 are expressed in heart and involved in myocardial I/R injury [36]. ROCK inhibition with fasudil or Y-27632 could significantly attenuate the injury induced by I/R in several* in vivo* models including mouse, rat, dog, and swine models. In this study, the results show that, compared with model group, TFR could obviously inhibit the increases of RhoA activity and ROCK1, ROCK2, and p-MLC protein expressions in WT rats, and this effect could also be achieved in UTR knockdown rats. However, the inhibitory percentage of TFR on RhoA activity and ROCK1, ROCK2, and p-MLC protein expressions in UTR knockdown rats decreased markedly in comparison with that in WT rats, which suggests that TFR inhibits the activation of RhoA/ROCK pathway induced by I/R injury, and this effect may be induced by its blocking UTR but not directly.

In conclusion, this study provides novel evidence that the protective effect of TFR on I/R injury may be correlated with its blocking UTR and subsequent inhibition of RhoA/ROCK signaling pathway.

## Figures and Tables

**Table 1:** Effect of TFR on combination of [^125^I]- hu-II and UTR.

Dose (×2.43 mg/L)	Inhibitory rate (*I*%)	IC_50_ (mg/L)
10^−3^	13.00	0.854
10^−2^	22.61	
10^−1^	35.74	
10^0^	51.25	
10^1^	82.45	
10^2^	90.99	
10^3^	95.07	
